# Sleep duration and anthropometric indices in an Iranian population: the Fasa PERSIAN cohort study

**DOI:** 10.1038/s41598-021-95796-9

**Published:** 2021-08-10

**Authors:** Mohammad Hosein Yazdanpanah, Mojtaba Farjam, Mohammad Mehdi Naghizadeh, Fariba Jedi, Kamand Mohebi, Reza Homayounfar

**Affiliations:** 1 Student Research Committee, Fasa University of Medical Sciences, Fasa, Iran; 2 Noncommunicable Diseases Research Center, Fasa University of Medical Sciences, Fasa, Iran; 3 National Nutrition and Food Technology Research Institute, Faculty of Nutrition Sciences and Food Technology, Shahid Beheshti University of Medical Sciences, Tehran, Iran

**Keywords:** Epidemiology, Obesity

## Abstract

Recent decades have seen a dramatic rise in the prevalence of obesity. While genetic factors can influence obesity, environmental factors and lifestyle may play important roles as well. Sleep can be regarded as one of these factors. This study aimed to examine sleep duration, as a potential risk factor for obesity in an Iranian population. In this cross-sectional study, the Fasa PERSIAN cohort study data was used and 10,136 subjects aged 35–70 were entered. Anthropometrics indices have been measured and the total body fat percentage (BFP) was obtained by Bio-Impedance Analysis. Also, physical activity and dietary intake have been recorded. Sleep duration was obtained and individuals categorized into two groups of “< 8” and “≥ 8” h of sleep. The mean age and sleep duration of the participants were 48.63 ± 9.57 years and 6.92 ± 1.62 h in the total population, respectively. All of the anthropometric indices were significantly higher in the “< 8 h of sleep” group than in the “≥ 8 h of sleep” group. Regarding BFP and fat mass index (FMI) the same results was seen (*p*-value < 0.05). Body mass index (BMI), Waist and hip circumferences (WC, HC), and waist-to-height ratio (WHtR) were in a significant negative association with night time sleep (*p*-value < 0.001), while these associations with daytime napping were positive (*p*-value < 0.001). After multi-variable adjusting, BMI, WC, HC, WHtR, and wrist circumference showed significant negative associations with 24-h sleep duration (*p*-value < 0.05). This study established the association between nocturnal, daytime napping, 24-h sleep duration and obesity parameters. Daytime napping was positively associated with obesity parameters and short 24-h sleep duration was associated with higher risk of overweight/obesity. These results indicate that insufficient sleep can be a screening indicator for an unhealthy lifestyle and poor health outcomes.

## Introduction

A significant increase has occurred in the prevalence of obesity in recent decades, and the World Health Organization (WHO) has named obesity as a global epidemic^1^. Obesity is associated with serious consequences such as the increased risk of diabetes, cardiovascular disease, arthritis, and cancer^2^. Although obesity can be affected by genetic factors, environmental factors, and lifestyle can also play significant roles. Sleep can be considered as one of the factors which may affect obesity^3^.

Chronic sleep deprivation has increased dramatically in the last half-century, as the average night-time sleep has decreased by 1.5 to 2 h. Today, more than 30% of employed US civilian adults sleep less than 6 h overnights^4^. Sleep can also change the overall energy balance in the body, increasing energy intake levels. Previous studies have shown that both acute sleep deprivation and chronic partial sleep deprivation can decrease serum leptin levels that leads to obesity^5^. As a strong appetite stimulant, ghrelin is higher in people who sleep less. Besides, short sleep duration may lead to obesity due to increased eating time^6^. Sleep deprivation can also cause fatigue in individuals, which may decrease physical activity levels^7,8^ and change in the energy intake and consumption (such as a physical activity)^3^. According to the aforementioned mechanisms and relationships, sleep deprivation may lead to weight gain and obesity.

Several previous study in different regions suggested the possible association of sleep duration and obesity in Japan^9^, Taiwan^10^ and the US^11,12^. But the factors that influence an individual's sleep length can differ across cultures and countries^13,14^. The relationship between sleep characteristics and health risks can be altered as a result of these variations. For example, the sleep duration of adults in the U.S. decreased more compared to adults in Finland in the past decade^15^. Similar studies in Iranian populations have yet to be conducted, and it would be particularly interesting to see whether the previous results could be replicated in a large Iranian community. Because many studies have shown inconsistent findings, there is still a knowledge gap due to ambiguity and a lack of research into the relationship between sleep duration and obesity. Also, this study would be the first study to investigate the relationship between sleep and obesity in an Iranian population using information related to anthropometric/body composition data.

Given the importance of adequate sleep-in individuals' physical and mental health, as well as the above-mentioned obesity-induced problems, this study aimed to examine sleep duration, as a potential risk factor for obesity, in the target population under controlling of the sleep/obesity factors.

## Methods

### Population

This sub-analysis used the data provided by the Fasa Cohort Study^16^. The Fasa Cohort Study is a part of the longitudinal PERSIAN Cohort Study designed to assess the risk factors for developing non-communicable diseases among the residents of a rural area, known as Sheshdeh (located in Fasa, Iran) with a total population of 41,000. The target population consisted of the residents of Sheshdeh in the 35–70 years-old age group (11,097 individuals). The data were collected from 2015 to 2016, and the participants were asked to sign a written informed consent form. In total, 986 individuals were excluded because of incomplete sleep and anthropometric information, history of chronic diseases such as hypothyroidism, hyperthyroidism, renal failure, cancer or sleep apnea disorder, consumption of medicines resulting in weight gain, and regular consumption of hypnotic sedative drugs. A total of 10,111 individuals (5539 women and 4572 men) were finally enrolled.

### Demographic data and anthropometrics measurement

Demographic data including age and gender were recorded in the questionnaire, which also cardiovascular diseases (CVD) history such as coronary heart diseases, myocardial infarction, and stroke. Each subject followed the protocol mentioned in reference 16. The participants’ heights and weights were measured and recorded by a trained person using a stadiometer with an accuracy of 0.1 cm (SECA 222 Stadiometer, Germany) and a calibrated digital scale with an accuracy of 0.1 kg (SECA 888 Digital Scale, Germany), respectively. The participants’ wrist (WrC), hip (HC), and waist circumference (WC) were measured using a special tape with an accuracy of 0.1 cm immediately after heights and weights measurement. Wrc was measured by asking subjects to keep their wrist anterior surface up; the tape measure's superior border was positioned just distal to the radial and ulnar bone prominences. WC was measured in the thinnest part of the waist between the tenth rib and the iliac crest. The maximum HC was also measured in the standing position.

All measurements were done without any clothes in bare foot condition. Also, waist to hip (WHR), and waist to height ratios (WHtR) were calculated by dividing the waist to hip and height in cm respectively.

### Socioeconomic status index

A principal component analysis (PCA) method was gen to generate a socioeconomic status (SES) index of respondents in the PERSIAN Cohort Study^17^. The dataset's available data on infrastructure facilities (source of drinking water, sanitation facility), housing condition (e.g., number of rooms, type of home ownership), and ownership of a variety of durable assets (e.g., dishwasher, vehicle, television), as well as education level, were used to create the SES variable for each participant.

### Body composition

Bioelectric impedance analysis (BIA) was used for measuring body composition using the Tanita BC-418 MA Segmental Body Composition Analyzer (Tanita, Japan). This is a single-frequency BIA device with eight polar electrodes and a single-point load cell weighing system in the scale platform that can provide separate body mass readings for various body segments including the right arm, left arm, trunk, right leg, and left leg. The impedance across the subject's tissues is determined with receiver electrodes after a predefined signal is passed through injector electrodes. All measurements are performed at 50 kHz with a steady current of 0.8 mA sine wave. FM percent is calculated using an algorithm that takes into account impedance, age, and height. The amount of fat mass, fat-free mass, and fat percentage in hands, trunk, legs, and the total body were reported separately. Also, the fat mass index (FMI) was calculated by dividing fat mass by the square of height in meters^18^. Among all individuals in the Fasa PERSIAN Cohort study, only 4661 subjects had the BIA body composition data which all of them was used in this study.

### Sleep duration

Using the first two questions Pittsburgh Sleep Quality questionnaire^19^ (When do you usually go to bed at night? “___” and When do you usually wake up in the morning? “___”) the trained person collected information about the participants’ sleep habits on the workdays. Moreover, sleep latency has been questioned from subjects and subsided from time in bed of subjects for having a better estimation of sleep duration. The same items were questioned about the weekends and sleep duration was calculated in hours by subtracting wake-up time from bedtime in both workdays and weekends. A mean of night sleep duration per day was calculated according to the participants answers. Also, regarding the napping time, the duration of the daytime napping was asked in hour and min in both workdays and weekends and an average of napping time was calculated. Finally, by adding average night sleep duration and average napping time, sleep duration per 24 h was calculated. According to the National Sleep Foundation, sleeping less than 8 h is considered insufficient^20^.

### Food frequency questionnaire (FFQ)

Nutritional information was measured using the modified FFQ, which examines the eating habits of individuals over a year. The participants were asked to report their food consumption program on a daily, weekly, monthly, or annual basis over the last year. The consumed foods were converted from household measures to grams. This modified FFQ included food items which was designed based on the information provided by experienced nutritionists familiar with the local diet of Iran. For the most part, the USDA food composition table (FCT) was used (USDA, Release 11, 1994). The Iranian food composition table was consulted for some products such as bread, vetch, pepper green, wild plum, mint, sweet canned cherry, and sour cherry^21^. This population-specific FFQ validation has been studied previously^22^. Finally, the total energy intake per day was calculated and reported.

### Physical activity (PA)

The International Physical Activity Questionnaire (IPAQ) was used to calculate physical activity levels. This 20-item questionnaire can measure routine physical activities of rural Iranians. The amount of each activity in hours and minutes was determined; the MET-value of each activity was multiplied by its duration, and total MET score was calculated.

### Statistics

All variables are reported as mean ± standard deviation, number (percentage). For comparison between two groups, the independent-samples t-test was used and for categorical variables, the chi-square test was performed. In 3 linear regression models, we performed our final analysis to minimize the effect of confounding variables including age, psychical activity, and energy intake. In model 1 without any variables, in model 2 with age, cardiovascular diseases(CVD) history^23^, socio-economic index, in model 3 with age, CVD history, socio-economic index and physical activity (MET score), dietary intake (energy intake Kcal/day) we adjusted our results. For calculating the odds ratio (OR) and 95% Confidence Interval (CI), logistic regression was used. Categorization of anthropometric and body composition variables to binary outcomes was done using previously published cut-off points^24–26^ and above adjustment method. In another linear regression analysis night and daytime sleep duration were regressed on anthropometrics and body fat indices. Also, our analysis was stratified by gender due to physiological different and different patterns of sleep. A significance level of *p*-value < 0.05 was considered, and all analyses were performed using IBM SPSS Statistics, version 23 (IBM Corp., Armonk, N.Y., USA). For forest plot graphs, Prism version 8.00 (GraphPad Software, La Jolla, California, USA) was used.

### Ethical statement

The study protocol was following the Helsinki Declaration and confirmed by the Ethics Committee of Fasa University of Medical Sciences (Approval Code: IR.FUMS.REC.1398.009). The participants were informed about the research objectives and the written informed consent was obtained from the subjects before starting the survey.

## Results

In a total of 10,111 subjects, the mean age of the participants was 48.61 ± 9.61 years for men and 48.64 ± 9.54 years for women; there was no significant difference between men and women in this regard (*p* = 0.841). The mean duration of sleep for men and women was 6.82 ± 1.65 and 6.97 ± 1.59 h, respectively, with a significant difference (*p* < 0.001). The participants were divided into two groups of “< 8 h of sleep” and “≥ 8 h of sleep” which included 6817 persons (67.5%) and 3294 persons (32.5%), respectively. The means of age in the “< 8 h of sleep” group were significantly higher in both genders. Moreover, SES of subjects with under 8 h of sleep was significantly higher than the other group.

In both genders, anthropometric indices, including BMI, WC, HC, WrC, Waist to hip ratio (WHR), and Waist to height ratio (WHtR) were significantly higher in the “< 8 h of sleep” group (*p* < 0.05). Table 1 presents the mean ± SD of demographic variables, anthropometric indices and body composition data according to gender. Moreover, Table S1 represent the means of anthropometric and body composition data by a different strata of sleep duration. Mostly, subjects with 4–6 h of sleep duration had the highest mean of anthropometric value among other groups.

**Table 1:** The comparison of anthropometric and body composition data between sleep groups according to gender.

Variables	Male			Female		
	Sleep duration		*p*-value	Sleep duration		*p*-value
	Under 8 h	8 h and more		Under 8 h	8 h and more	
Sample size	3163	1409		3651	1888	
Age (year)	48.8 ± 9.5	48.1 ± 9.9	**0.024**	49.2 ± 9.4	47.6 ± 9.7	** < 0.001**
Socio-economic index	0.724 ± 2.499	0.17 ± 2.266	** < 0.001**	− 0.363 ± 1.716	− 0.646 ± 1.571	** < 0.001**
MET score	45.46 ± 14.53	44.61 ± 13.85	0.067	39.15 ± 6.65	37.04 ± 6.64	** < 0.001**
Energy (kcal/day)	3627.6 ± 1246.9	3594.3 ± 1216.6	0.401	2800.7 ± 900	2837.6 ± 908.5	0.149
BMI (kg/m^2^)	24.42 ± 4.45	23.69 ± 4.26	** < 0.001**	27.05 ± 4.83	26.49 ± 4.75	** < 0.001**
WC (cm)	89.97 ± 11.31	88.49 ± 10.89	** < 0.001**	96.68 ± 11.45	95.15 ± 11.50	** < 0.001**
HC (cm)	97.89 ± 7.72	96.74 ± 7.52	** < 0.001**	101.56 ± 9.41	100.71 ± 9.42	**0.001**
WrC (cm)	17.34 ± 1.24	17.14 ± 1.20	** < 0.001**	16.30 ± 1.27	16.20 ± 1.25	**0.004**
WHR	0.917 ± 0.65	0.912 ± 0.62	**0.040**	0.95 ± 0.061	0.94 ± 0.062	** < 0.001**
WHtR	0.53 ± 0.66	0.52 ± 0.64	** < 0.001**	0.62 ± 0.074	0.61 ± 0.075	** < 0.001**

Sample size	1528	627		1676	830	
**Arms**						
Fat mass (kg)	1.47 ± 0.78	1.35 ± 0.70	**0.001**	2.60 ± 1.24	2.47 ± 1.20	**0.009**
Fat-free mass (kg)	6.21 ± 1.17	6.01 ± 1.06	** < 0.001**	4.31 ± 0.60	4.28 ± 0.62	0.145
Fat mass (%)	36.31 ± 11.52	34.73 ± 11.60	**0.004**	71.35 ± 17.71	69.21 ± 17.79	**0.005**
**Legs**						
Fat mass (kg)	4.13 ± 2.07	3.81 ± 1.99	**0.001**	9.88 ± 2.82	9.57 ± 2.75	**0.008**
Fat-free mass (kg)	18.79 ± 2.85	18.39 ± 2.57	**0.003**	14.11 ± 1.81	13.99 ± 1.76	0.136
Fat mass (%)	34.36 ± 11.53	32.57 ± 12.01	**0.001**	81.00 ± 9.89	79.81 ± 9.85	**0.005**
**Trunk**						
Fat mass (kg)	9.09 ± 4.73	8.38 ± 4.47	**0.001**	10.97 ± 4.52	10.39 ± 4.35	**0.002**
Fat-free mass (kg)	30.22 ± 4.00	29.48 ± 3.81	** < 0.001**	24.27 ± 2.56	24.14 ± 2.58	0.227
Fat mass (%)	21.90 ± 8.38	20.93 ± 8.43	**0.015**	29.92 ± 8.11	28.87 ± 8.22	**0.002**
**Total**						
Fat mass (kg)	14.66 ± 7.50	13.50 ± 7.06	**0.001**	23.33 ± 8.43	22.40 ± 4.84	**0.003**
Fat-free mass (kg)	55.23 ± 7.82	53.89 ± 7.20	** < 0.001**	42.68 ± 4.84	42.40 ± 4.84	0.166
Fat mass (%)	19.91 ± 7.14	18.98 ± 7.19	**0.006**	34.37 ± 6.80	33.50 ± 6.86	**0.003**
**FMI (kg/m**^**2**^**)**	5.12 ± 2.57	4.72 ± 2.46	**0.001**	9.67 ± 3.37	9.25 ± 3.32	**0.004**

*BMI* body mass index, *WC* waist circumferences, *HC* hip circumferences, *WrC* wrist circumferences, *WHR* waist to hip ratio, *WHtR* waist to height ratio, *FMI* fat mass index.

Bold values are less than 0.05 and are statistically significant.

**Figure 1:**
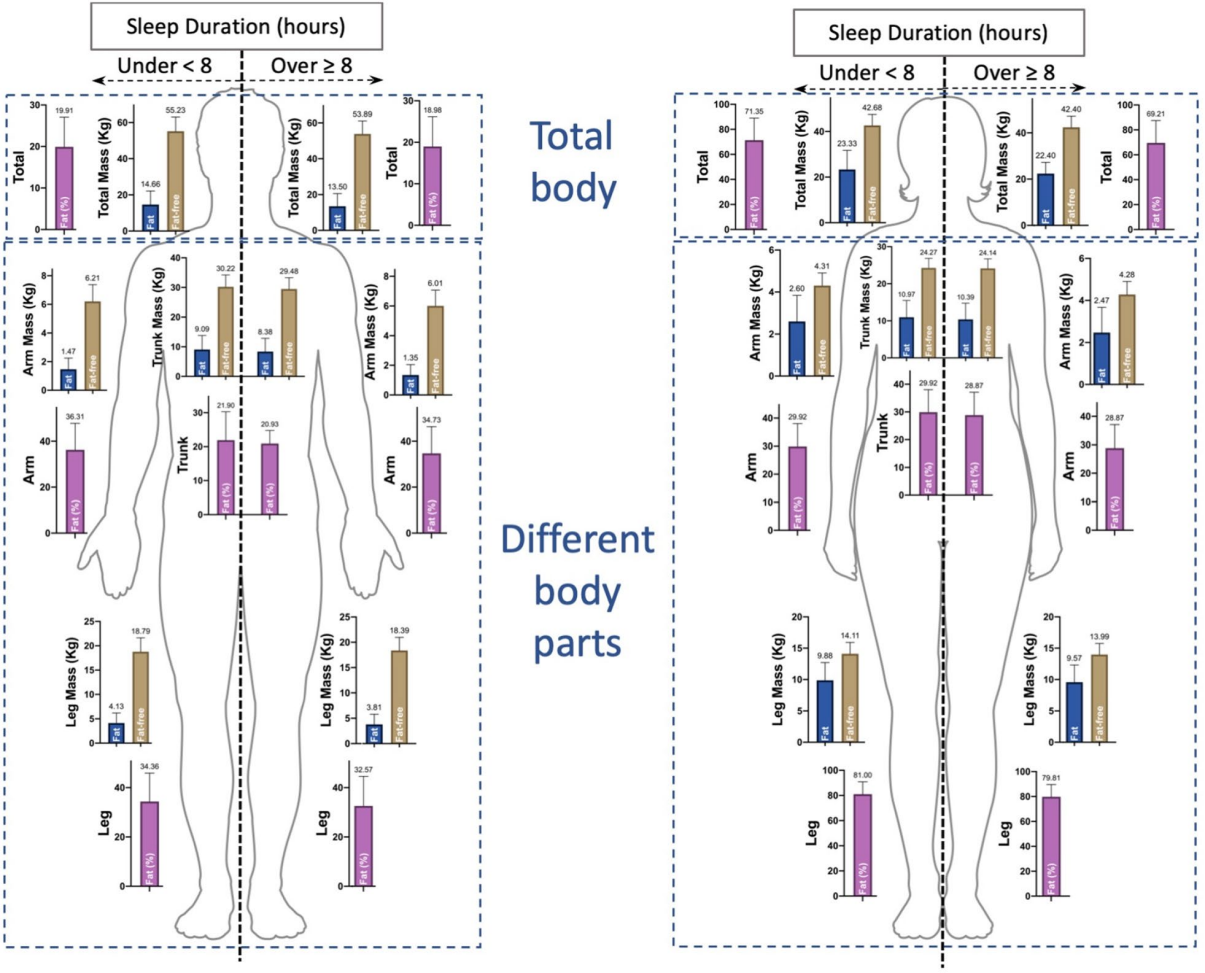
The schematic graphs of body fat composition data in total and different body parts in both gender.

Body composition indices of arms, legs, trunk, and the whole body in the two sleep groups are compared in Table 1. All these indices were higher in the insufficient sleep group. In both genders, the amount of fat (kg and percentage) was significantly higher in the insufficient sleep group (*p* < 0.01). There was a significant difference between men in the two groups in terms of the amount of fat-free mass (*p* < 0.001); however, no significant difference was observed in women in this respect (*p* > 0.05). Besides, the FMI was significantly higher in men and women in the " < 8 h of sleep" group than in those in the " ≥ 8 h of sleep" group (*p* < 0.01). Figure 1 shows a schematic representation of the body composition data of the results of Table 1.

In Table 2, the linear association of the anthropometric data and fat mass index with night and day sleep hour duration as continuous variables in both genders is reported. All of the standardized beta coefficient of the association of each anthropometric/body composition variable and night sleep duration was negative, while nap time coefficients were positive. The highest positive coefficients were related to BMI, HC, WC and nap time (0.111, *p*-value < 0.001) in males and BMI and nap time (0.111, *p*-value < 0.001) in females. Also, the highest negative coefficients were related BMI and night sleep (− 0.074, *p*-value < 0.001) in male, WHtR and night sleep (− 0.067, *p*-value < 0.001) in females. Also, the linear, quadratic and cubic association of anthropometric and body composition data with sleep hours according to gender has been reported in Table S2. BMI and HC have shown slight negative significant beta coefficients in the quadratic model in both genders. In the cubic models, WC and WHtR were in significant associations with sleep duration in both genders (*p*-value < 0.05). However, some of the beta coefficients such as WHtR in both genders were too small (b = 0.001).

**Table 2:** Linear regression model of anthropometric data, body fat percentage, and fat mass index with night and daytime sleep hour duration as continuous variables in both gender.

Dependent variable	Independent variable	Male				Female			
		B	SE (B)	Beta	*p*-value	B	SE (B)	Beta	*p*-value
BMI (kg/m^2^)	(Constant)	25.093	0.284		< 0.001	27.298	0.292		< 0.001
	Night sleep	− 0.003	0.001	− **0.074**	** < 0.001**	− 0.002	0.001	− **0.043**	**0.001**
	Daytime sleep	0.009	0.001	**0.111**	** < 0.001**	0.010	0.001	**0.108**	** < 0.001**
WC (cm)	(Constant)	91.008	0.721		< 0.001	98.293	0.698		< 0.001
	Night sleep	− 0.007	0.002	− **0.057**	** < 0.001**	− 0.007	0.002	− **0.057**	** < 0.001**
	Daytime sleep	0.023	0.003	**0.111**	** < 0.001**	0.016	0.003	**0.072**	** < 0.001**
HC (cm)	(Constant)	98.817	0.494		< 0.001	101.728	0.572		< 0.001
	Night sleep	− 0.005	0.001	− **0.066**	** < 0.001**	− 0.003	0.001	− **0.032**	**0.018**
	Daytime sleep	0.015	0.002	**0.111**	** < 0.001**	0.018	0.002	**0.098**	** < 0.001**
WrC (cm)	(Constant)	17.532	0.080		< 0.001	16.264	0.077		< 0.001
	Night sleep	− 0.001	0.000	− **0.068**	** < 0.001**	0.000	0.000	− 0.015	0.251
	Daytime sleep	0.002	0.000	**0.083**	** < 0.001**	0.002	0.000	**0.082**	** < 0.001**
WHR	(Constant)	0.919	0.004		< 0.001	0.966	0.004		< 0.001
	Night sleep	0.000	0.000	− 0.027	0.068	0.000	0.000	− **0.060**	** < 0.001**
	Daytime sleep	0.000	0.000	**0.071**	** < 0.001**	0.000	0.000	− 0.008	0.571
WHtR	(Constant)	0.539	0.004		< 0.001	0.636	0.005		< 0.001
	Night sleep	0.000	0.000	− **0.056**	** < 0.001**	− 0.000	0.000	− **0.067**	** < 0.001**
	Daytime sleep	0.000	0.000	**0.099**	** < 0.001**	0.000	0.000	**0.061**	** < 0.001**
Body fat (%)	(Constant)	51.589	0.843		< 0.001	48.371	0.760		< 0.001
	Night sleep	− 0.005	0.002	− **0.051**	**0.018**	− 0.001	0.002	− 0.010	0.603
	Day sleep	0.010	0.003	**0.059**	**0.005**	0.011	0.003	**0.068**	**0.001**
FMI (kg/m^2^)	(Constant)	6.699	0.303		< 0.001	8.347	0.334		< 0.001
	Night sleep	0.000	0.001	− 0.009	0.675	− 0.001	0.001	− 0.020	0.326
	Daytime sleep	− 0.002	0.001	− 0.028	0.196	0.001	0.001	0.017	0.413

Beta: Standardized regression coefficients (calculated as b_i_ × S_Xi_/S_Y_). *BMI* body mass index, *WC* waist circumferences, *HC* hip circumferences, *WrC* wrist circumferences, *WHR* waist to hip ratio, *WHtR* waist to height ratio, *FMI* fat mass index.

Bold values are less than 0.05 and are statistically significant.

The relationships of sleep duration with obesity parameters are shown in Table 3. In both genders, all variables of anthropometrics and body composition such as body fat percentage and FMI were inversely correlated with hours of sleep, and all had a statistically significant relationship except for the variables WHR and FMI in men, and WHR, BFP, and FMI in women. Among the results of the model 3, the highest significant beta coefficient among anthropometrics and body composition variables were related to BMI (b =  − 0.059) in men and WHtR (b =  − 0.064) in women. In men, BFP was the second place with (b =  − 0.056). In women, HC and WC both with (b =  − 0.062) were the second and third places.

**Table 3:** The linear regression models of anthropometric data, body fat percentage and fat mass index with 24-h sleep duration as a continuous variable in both gender.

Model	Dependent variable	Independent variable:	Male				Female			
			Unstandardized coefficients		Standardized coefficients	*p*-value	Unstandardized coefficients		Standardized coefficients	*p*-value
		Sleep duration	B	Std. error	Beta		B	Std. error	Beta	
Model 1	BMI (kg/m^2^)		− 0.205	0.04	− **0.077**	** < 0.001**	− 0.102	0.041	− **0.034**	**0.012**
Model 2			− 0.166	0.039	− **0.062**	** < 0.001**	− 0.102	0.041	− **0.034**	**0.012**
Model 3			− 0.157	0.038	− **0.059**	** < 0.001**	− 0.182	0.041	− **0.060**	** < 0.001**
Model 1	WC (cm)		− 0.406	0.100	− **0.060**	** < 0.001**	− 0.369	0.097	− **0.051**	** < 0.001**
Model 2			− 0.283	0.098	− **0.042**	**0.004**	− 0.250	0.098	− **0.035**	**0.010**
Model 3			− 0.263	0.097	− **0.039**	**0.007**	− 0.446	0.099	− **0.062**	** < 0.001**
Model 1	HC (cm)		− 0.316	0.069	− **0.068**	** < 0.001**	− 0.136	0.080	− 0.023	0.088
Model 2			− 0.258	0.068	− **0.055**	** < 0.001**	− 0.221	0.079	− **0.037**	**0.005**
Model 3			− 0.239	0.067	− **0.051**	** < 0.001**	− 0.368	0.080	− **0.062**	** < 0.001**
Model 1	WrC (cm)		− 0.052	0.011	− **0.070**	** < 0.001**	− 0.007	0.011	− 0.008	0.545
Model 2			− 0.042	0.011	− **0.057**	** < 0.001**	− 0.008	0.011	− 0.010	0.444
Model 3			− 0.038	0.011	− **0.051**	** < 0.001**	− 0.023	0.011	− **0.029**	**0.035**
Model 1	WHR		− 0.001	0.001	− 0.028	0.054	− 0.002	0.001	− **0.061**	** < 0.001**
Model 2			0.000	0.001	− 0.010	0.479	0.000	0.000	− 0.010	0.456
Model 3			0.000	0.001	− 0.009	0.512	− 0.001	0.001	− 0.024	0.065
Model 1	WHtR		− 0.002	0.001	− **0.058**	** < 0.001**	− 0.003	0.001	− **0.062**	** < 0.001**
Model 2			− 0.002	0.001	− **0.040**	**0.005**	− 0.002	0.001	− **0.037**	**0.006**
Model 3			− 0.002	0.001	− **0.039**	**0.006**	− 0.003	0.001	− **0.064**	** < 0.001**
Model 1	Body fat (%)		− 0.287	0.118	− **0.052**	**0.016**	− 0.017	0.107	− 0.003	0.876
Model 2			− 0.308	0.119	− **0.056**	**0.009**	− 0.145	0.108	− 0.027	0.181
Model 3			− 0.310	0.119	− **0.056**	**0.009**	− 0.161	0.112	− 0.030	0.148
Model 1	FMI (kg/m^2^)		− 0.017	0.043	− 0.008	0.692	− 0.042	0.047	− 0.018	0.369
Model 2			− 0.005	0.042	− 0.003	0.903	0.01	0.047	0.004	0.833
Model 3			− 0.006	0.043	− 0.003	0.889	− 0.035	0.049	− 0.015	0.474

Standardized regression coefficients calculated as b_i_ × S_Xi_/S_Y_. *BMI* body mass index, *WC* waist circumferences, *HC* hip circumferences, *WrC* wrist circumferences, *WHR* waist to hip ratio, *WHtR* waist to height ratio, *FMI* fat mass index. Model 1: unadjusted, Model 2: Adjusted with age, CVD history, socio-economic index, Model 3: adjusted with age, CVD history, socio-economic index and physical activity (MET score), dietary intake (energy intake Kcal/day).

Bold values are less than 0.05 and are statistically significant.

**Figure 2:**
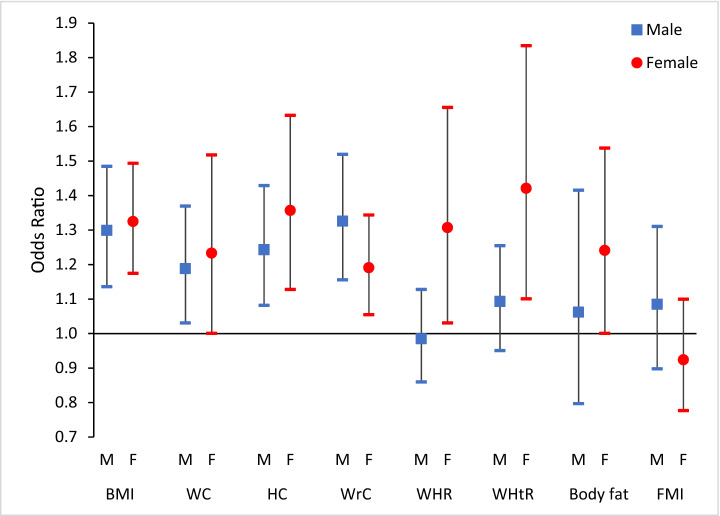
The odds ratios of multivariate model of binary logistic regression between sleep and anthropometric data, body fat percentage and fat mass index as binary outcomes in both genders. BMI = Body Mass Index, WC = Waist Circumferences, HC = Hip Circumferences, WrC = Wrist Circumferences, WHR = Waist to Hip Ratio, WHtR = Waist to Height Ratio, FMI = Fat Mass Index. Multivariate model: adjusted with age, physical activity and dietary intake. Sleep considered as a binary variable in the model (Sleep hours < 8 = 1).

Figure 2 shows the odds ratios in a binary logistic model between sleep and anthropometric data, body fat percentage, and fat mass index, all as binary outcomes in both genders. BMI, WC, HC, and WrC in males showed significant ORs in the final multi-variable adjusted model. In females, in addition to the above-mentioned variables, WHR, WHtR, and BFP had significant ORs in the third model. Highest OR in males was 1.326 (1.156–1.520, *p*-value < 0.001) which was related to WrC and in females it was 1.421 (1.101–1.835, *p*-value < 0.05) which was related to WHtR. ORs was higher in female than the male with one exception which was WrC, and also significant levels in males were stronger. Table S3 in the supplementary file represents all the ORs in 3 models of binary logistic between sleep and anthropometric data, body fat percentage, and fat mass index in both genders. Moreover, Table S4 representing the intercorrelation coefficients between anthropometric and body composition data.

## Discussion

### Main findings

The main findings of the present study were: a) All of the anthropometric indices were significantly higher in the “< 8 h of sleep” group than in the “≥ 8 h of sleep” group. Regarding BFP and FMI the same results was seen. b) BMI, WC, HC, and WHtR were in a significant negative association with night time sleep, while these associations with nap time sleep duration were positive in both genders. c) After adjusting for age, CVD history, socio-economic index and physical activity, dietary intake, BMI, WC, HC, WrC and WHtR showed significant negative associations with 24-h sleep duration in both genders. To the best of our knowledge, this is the first study to investigate the relationship between sleep and obesity in an Iranian population using information related to different body parts’ fat percentage adjusted by the numerous obesity/sleep-related variables.

### Sleep duration and physical activity

Chronic partial sleep deprivation leads to fatigue, which in turn reduces physical activity levels. In this study, subjects with under 8 h of sleep reported higher MET score which was only significant in women. Previous studies have shown that shorter sleep duration is associated with increased television viewing and decreased participation in sports^27^. Short sleepers spend more time watching TV, and the number of hours spent watching TV is associated with the amount of weight gained^28^. A report from the UK shows increased bedtime TV viewing among the target group, which is associated with reduced sleep duration^29^. Accelerometers were used in another study, which results showed that short sleepers significantly spend more time on low-mobility activities than those who sleep a minimum of 8 h a day. These results remained significant after adjusting for BMI, indicating that the relationship between inactivity and sleep duration is independent of obesity^6^. Although these studies have been conducted on adolescents and children, similar results are also expected to be obtained for adults. Similar to our findings, in a study conducted on adults in the United States, it was found that short-term sleep loss reduces spontaneous daytime activity, leading to lower intensities of physical activity under free-living conditions^30^. The results of some studies are not in line with the present results. For example, in an interventional study, two groups of volunteers slept for 5.5 and 8.5 h per day over 14 days, and the results showed an insignificant difference between two groups in daily physical activity levels^31^.

### Sleep duration and food intake habits

Repeated sleep restrictions can disrupt the amount, distribution, and composition of human food intake. In general, we have more time to eat when we sleep less. In this study, nine main categories of foods including cereals, dairy, vegetables, fruits, salt, oil, beans, meat, and sugar were examined. Based on the results, the total intake of calories per day for did not show any significant difference in both genders. Another study showed that short sleepers are less likely to have a good diet compared with those who sleep more than 8 h a day. Garaulet et al. found that people with an insufficient amount of sleep eat fewer fruits, vegetables, fish, milk, cereals, and breakfast, but more unhealthy foods such as pizza, hamburgers, and pasta compared to others^6^. Although no significant difference was observed between the two sleep groups in terms of energy intake, studies have suggested that people are more likely to eat food, especially snacks, when they sleep less^31,32^.

### Daytime napping duration and anthropometric data

The association of daytime napping with obesity has been envaulted in several previous studies. Chen et al. studied 1267 Chinese adults and reported that the frequency of daytime napping was negatively associated with BMI and short nocturnal sleep duration was associated with higher risk of overweight/obesity^33^. In our study, BMI had the highest significant positive association among other anthropometric data with daytime sleep duration. It has been also suggested that obesity was associated with the incidence and persistence of daytime sleepiness, while weight loss was associated with its remission^34^. There are also other studies which was in a line with our findings^35,36^. Another study in the US reported that nappers have higher values of WHR in comparison to non-nappers^37^. Also, in our study WHR showed a positive association with daytime sleep duration in men. Moreover, longer napping time has been showed that is associated with type 2 diabetes^38,39^, CVD^40^, and non-alcoholic fatty liver disease^41^.

### Sleep duration and anthropometric data

In both genders, all anthropometric indices were higher in the “< 8 h of sleep” group which was in a line with different studies from Sweden^42^, Canada^32^, and the US. The National Health and Nutrition Examination Survey (NHANES) data showed that the highest and the lowest BMI was observed in people with the shortest and longest hours of sleep, respectively^43^. In both genders, FMI was significantly lower in the “≥ 8 h of sleep” group. According to previous studies, poor sleep quality was associated with increased FMI in adults, because a reduction in sleep efficiency (from 90 to 85%) would lead to an increase in FMI (from 5.3 to 6.5 kg/m^2^ in women and from 3.6 to 4.8 kg/m^2^ in men)^44^. The above studies confirm the present results in terms of significant increases in anthropometric indices of people with less sleep. Differences in the result of previous literature and this study may be due to differences in sampling, gender, ethnicity, age, classification method, sample size, cultural and geographical differences.

We examined 3 models to determine whether factors such as age, CVD history, socio-economic index and physical activity (MET score), dietary intake (energy intake Kcal/day) affect the relationship between sleep and obesity. In men, the beta coefficient of the multi-variable adjusted model of BMI was the highest. However, in a previous study, the correlation between BMI and sleep duration hours was − 0.070 in the total population which was higher than our results^6^. Beta coefficients for WC remained significant after adjusting, but it was higher in women comparing to men. The results of several articles in the UK indicate that there is a significant negative relationship between sleep duration and WC^45^ which was in line with our results. Regarding the HC variable, the beta coefficient in the first model of men was significant while in the female it was not significant. Interestingly, after multivariable adjustment, the beta coefficient in females became significant and higher than the males’ beta coefficient. It is noteworthy that beta coefficients of BMI, WC, WHtR, and HC were higher in females than males which indicates that shorter sleep hours may have a higher effect in increasing these indices in females. In the case of WrC, the beta coefficients after adjustment were weakened but remained significant in males while there was only a significant relationship in females just in model 3. In previously published articles, it has been declared that WrC may be a novel predictor for hypertension, cardiovascular disease^46^, and diabetes, and prediabetes^26^. Our results suggested that sleep hours may affect WrC and other anthropometric indices which should be considered in further studies as an independent factor. There was not any significant relationship between WHR and sleep hours in final model. Moreover, regarding the variable of WHtR, the relationship remained significant after multi-variable adjusting. It should be mention that the beta coefficient for WHtR in women was the highest beta coefficient among other variables in both genders. In the BFP variable in men, we observed a slight increase in beta coefficients, while the relationship remained significant. Also, the BFP’s final beta coefficient was the second rank among other indices in men while it was not significant in women.

### Possible mechanisms

The association between sleep duration and obesity may be due to changes in the levels of some neuropeptides involved in appetite-regulation^32,47,48^. Sleep deprivation can cause neuro-hormonal disorders resulting in increased calorie intake levels^48^. Hormones also play a key role in this mechanism. Leptin and ghrelin are two of these hormones, which play different roles in appetite-regulation^49,50^. Adiponectin, which is secreted by adipose tissues, also plays a major role in the energy balance as a mediator of metabolism^51,52^.

## Limitations

The current paper is not free of limitations this was a cross-sectional study; therefore, we cannot confidently specify the relationships of hours of sleep with obesity parameters. To suggest a cause-and-effect method, close observation must be made during the analysis of the Fasa PERSIAN cohort study at longer follow-up intervals, which can add to the importance of the research. Also, the current research was based on the individuals' self-report and therefore the data were subjective and may be prone to substantial recall bias. However, Sankai et al. have suggested that self-report sleep is valid compared to actigraphy^53^. In the current research, factors affecting the duration of sleep, and other quality factors, were not assessed; rather, the emphasis was focused solely on the amount of sleep. There was no recorded cause for the short period of sleep among individuals in the data; it may be the product of psychological or physical disorders, or just being tired from overwork. These variables are recommended to be considered during the follow-ups. The current study examined a rural population; a related study is suggested to be held in an urban community to determine any potential variations.

## Conclusion

The present study suggests that anthropometric parameters such as BMI, WC, HC, WrC, WHtR are in inverse relationships with sleep hours. In women, anthropometric parameters such as BMI, WC, HC, and WHtR are more likely to increase with decreasing sleep hours. Finally, in the linear regression models, BFP was inversely associated with hours of sleep in men, while no significant correlation was found between this variable and hours of sleep in women. These findings suggest that a lack of sleep may be a warning sign of an unhealthy lifestyle and poor health effects.

## Supplementary Information

Supplementary Tables.

## Abbreviations

BMI: Body mass index
BFP: Body fat percentage
BIA: Bioelectric impedance analysis
FCT: USDA food composition table
FFQ: Food frequency questionnaire
FMI: Fat mass index
HC: Hip circumference
IPAQ: International physical activity questionnaire
MET: Metabolic equivalent of task
PCA: Principal component analysis
SES: Socioeconomic status
WC: Waist circumference
WHR: Waist to hip ratios
WHtR: Waist to height ratios
WrC: Wrist circumference
