# Supplementary material for: Sleep duration and anthropometric indices in an Iranian population: the Fasa PERSIAN cohort study
Source: Sci Rep. 2021 Aug 10;11:16249. doi: 10.1038/s41598-021-95796-9 (PMC8355308; doi:10.1038/s41598-021-95796-9)
Supplement: Supplementary file 1 — Supplementary Tables. [file 41598_2021_95796_MOESM1_ESM.docx]

**Supplementary File**

**Sleep duration and anthropometric indices in an Iranian population: The Fasa PERSIAN Cohort Study**

Mohammad Hosein Yazdanpanah^1,2^, Mojtaba Farjam^2^, Mohammad Mehdi Naghizadeh^2^, Fariba Jedi^1^, Kamand Mohebi^1^, Reza Homayounfar^2,3^*

1- Student Research Committee, Fasa University of Medical Sciences, Fasa, Iran

2- Noncommunicable Diseases Research Center, Fasa University of Medical Sciences, Fasa, Iran

3- National Nutrition and Food Technology Research Institute, Faculty of Nutrition Sciences and Food Technology, Shahid Beheshti University of Medical Sciences, Tehran, Iran

**Running title:** *Sleep duration and obesity*

**Keywords:** Sleep Duration, Obesity, Anthropometrics, Fat mass

**Conflict of Interest**: Authors have no competing interest in the results of the article

**Total Word Count without references:** 4191 (abstract + main text)

**Number of Tables**: 3

**Number of Figures**: 2

***Corresponding Author:**

Reza Homayounfar, MPH, PhD

National Nutrition and Food Technology Research Institute, Faculty of Nutrition Sciences and Food Technology, Shahid Beheshti University of Medical Sciences, Tehran, Iran.

E-mail: [r_homayounfar@yahoo.com](mailto:r_homayounfar@yahoo.com), homayounfar@sbmu.ac.ir

Tel number: +989125140840

Table S1-a. The means of anthropometric and body composition data between sleep groups (<4, 4-6, 6-8, 8-10, >10) according to gender

|  |  | | **Sleep** | | | | |
| --- | --- | --- | --- | --- | --- | --- | --- |
|  |  |  | **<4** | **4-6** | **6-8** | **8-10** | **>10** |
| **Male** | **Anthropometric** | **N** | **159** | **804** | **2912** | **633** | **59** |
|  |  | BMI (kg/m2) | 24.47 ± 4.50 | 24.59 ± 4.46 | 24.26 ± 4.42 | 23.45 ± 4.26 | 23.33 ± 3.48 |
|  |  | WC (cm) | 89.859 ± 11.399 | 90.524 ± 11.533 | 89.551 ± 11.121 | 88.162 ± 11.242 | 88.005 ± 8.515 |
|  |  | HC (cm) | 97.910 ± 7.750 | 98.238 ± 7.854 | 97.612 ± 7.668 | 96.404 ± 7.492 | 95.624 ± 6.657 |
|  |  | WrC (cm) | 17.457 ± 1.258 | 17.376 ± 1.216 | 17.284 ± 1.238 | 17.136 ± 1.250 | 17.075 ± 1.089 |
|  |  | WHR | 0.915 ± 0.062 | 0.919 ± 0.065 | 0.916 ± 0.065 | 0.912 ± 0.065 | 0.920 ± 0.051 |
|  |  | WHtR | 0.531 ± 0.067 | 0.536 ± 0.068 | 0.530 ± 0.066 | 0.522 ± 0.066 | 0.524 ± 0.050 |
|  | **Body composition** | **N** | **76** | **386** | **1413** | **287** | **21** |
|  |  | FMI (kg/m2) | 6.427 ± 3.243 | 6.376 ± 3.023 | 6.542 ± 3.210 | 6.567 ± 3.377 | 5.592 ± 2.806 |
|  |  | Body fat (%) | 51.597 ± 10.908 | 50.436 ± 8.661 | 50.086 ± 8.938 | 49.915 ± 8.416 | 45.49 ± 8.767 |
| **Female** | **Anthropometric** | **N** | **141** | **866** | **3580** | **891** | **66** |
|  |  | BMI (kg/m2) | 26.27 ± 4.53 | 27.21 ± 4.93 | 26.95 ± 4.79 | 26.30 ± 4.80 | 26.62 ± 4.90 |
|  |  | WC (cm) | 95.713 ± 11.367 | 97.333 ± 11.810 | 96.241 ± 11.291 | 94.804 ± 11.858 | 95.686 ± 11.856 |
|  |  | HC (cm) | 100.072 ± 8.627 | 101.717 ± 9.633 | 101.452 ± 9.420 | 100.383 ± 9.286 | 100.121 ± 9.700 |
|  |  | WrC (cm) | 16.082 ± 1.14 | 16.269 ± 1.407 | 16.306 ± 1.232 | 16.193 ± 1.3 | 16.164 ± 1.374 |
|  |  | WHR | 0.955 ± 0.064 | 0.956 ± 0.064 | 0.948 ± 0.061 | 0.943 ± 0.064 | 0.955 ± 0.058 |
|  |  | WHtR | 0.616 ± 0.075 | 0.628 ± 0.077 | 0.618 ± 0.074 | 0.608 ± 0.077 | 0.617 ± 0.078 |
|  | **Body composition** | **N** | **69** | **432** | **1566** | **386** | **25** |
|  |  | FMI (kg/m2) | 7.939 ± 3.698 | 8.254 ± 4.102 | 8.050 ± 3.685 | 8.028 ± 3.710 | 8.045 ± 4.227 |
|  |  | Body fat (%) | 46.677 ± 7.637 | 48.511 ± 8.62 | 48.639 ± 8.714 | 47.974 ± 8.274 | 50.492 ± 8.488 |

BMI= Body Mass Index, WC= Waist Circumferences, HC= Hip Circumferences, WrC= Wrist Circumferences, WHR= Waist to Hip Ratio, WHtR= Waist to Height Ratio, FMI=Fat Mass Index

Table S1-b. The means of anthropometric and body composition data between sleep groups (<6, 6-8, >8) according to gender

|  |  | | **Sleep** | | |
| --- | --- | --- | --- | --- | --- |
|  |  |  | **<6** | **6-8** | **>8** |
| **Male** | **Anthropometric** | **N** | **960** | **2912** | **692** |
|  |  | BMI (kg/m2) | 24.57 ± 4.47 | 24.26 ± 4.42 | 23.44 ± 4.20 |
|  |  | WC (cm) | 90.414 ± 11.508 | 89.551 ± 11.121 | 88.149 ± 11.031 |
|  |  | HC (cm) | 98.184 ± 7.834 | 97.612 ± 7.668 | 96.338 ± 7.423 |
|  |  | WrC (cm) | 17.389 ± 1.223 | 17.284 ± 1.238 | 17.131 ± 1.236 |
|  |  | WHR | 0.919 ± 0.065 | 0.916 ± 0.065 | 0.913 ± 0.064 |
|  |  | WHtR | 0.535 ± 0.067 | 0.530 ± 0.066 | 0.522 ± 0.065 |
|  | **Body composition** | **N** | **460** | **1413** | **308** |
|  |  | FMI (kg/m2) | 6.384 ± 3.056 | 6.542 ± 3.210 | 6.501 ± 3.346 |
|  |  | Body fat (%) | 50.627 ± 9.067 | 50.086 ± 8.938 | 49.613 ± 8.499 |
| **Female** | **Anthropometric** | **N** | **1007** | **3580** | **957** |
|  |  | BMI (kg/m2) | 27.075 ± 4.883 | 26.949 ± 4.789 | 26.324 ± 4.809 |
|  |  | WC (cm) | 97.11 ± 11.76 | 96.24 ± 11.29 | 94.86 ± 11.85 |
|  |  | HC (cm) | 101.487 ± 9.512 | 101.452 ± 9.420 | 100.365 ± 9.310 |
|  |  | WrC (cm) | 16.243 ± 1.374 | 16.306 ± 1.232 | 16.191 ± 1.304 |
|  |  | WHR | 0.956 ± 0.064 | 0.948 ± 0.061 | 0.944 ± 0.064 |
|  |  | WHtR | 0.626 ± 0.077 | 0.618 ± 0.074 | 0.609 ± 0.077 |
|  | **Body composition** | **N** | **571** | **1566** | **411** |
|  |  | FMI (kg/m2) | 8.211 ± 4.046 | 8.050 ± 3.685 | 8.029 ± 3.738 |
|  |  | Body fat (%) | 48.258 ± 8.508 | 48.639 ± 8.714 | 48.127 ± 8.298 |

BMI= Body Mass Index, WC= Waist Circumferences, HC= Hip Circumferences, WrC= Wrist Circumferences, WHR= Waist to Hip Ratio, WHtR= Waist to Height Ratio, FMI=Fat Mass Index

Table S2. The linear, quadratic and cubic association of anthropometric data, body fat percentage, and fat mass index with sleep hours according to gender

| Dependent variable | Sleep hours | Male | | | | | | Female | | | | | |
| --- | --- | --- | --- | --- | --- | --- | --- | --- | --- | --- | --- | --- | --- |
|  |  | Linear | | Quadratic | | Cubic | | Linear | | Quadratic | | Cubic | |
|  |  | Coef. | P-value | Coef. | P-value | Coef. | P-value | Coef. | P-value | Coef. | P-value | Coef. | P-value |
| BMI (kg/m2) | Constant | 25.600 | <0.001 | 24.215 | <0.001 | 22.779 | <0.001 | 27.575 | <0.001 | 26.008 | <0.001 | 24.674 | <0.001 |
|  | b(x) | -0.205 | **<0.001** | 0.251 | 0.171 | 1.105 | **0.010** | -0.102 | **0.012** | 0.394 | 0.051 | 1.133 | **0.022** |
|  | b(x^2^) |  |  | -0.035 | **0.011** | -0.182 | **0.008** |  |  | -0.037 | **0.012** | -0.159 | **0.037** |
|  | b(x^3^) |  |  |  |  | 0.008 | **0.029** |  |  |  |  | 0.006 | 0.102 |
| WC (cm) | Constant | 92.297 | <0.001 | 89.674 | <0.001 | 86.144 | <0.001 | 98.734 | <0.001 | 95.909 | <0.001 | 91.282 | <0.001 |
|  | b(x) | -0.406 | **<0.001** | 0.457 | 0.327 | 2.556 | **0.020** | -0.369 | **<0.001** | 0.525 | 0.276 | 3.090 | **0.009** |
|  | b(x^2^) |  |  | -0.066 | 0.058 | -0.427 | **0.014** |  |  | -0.067 | 0.058 | -0.489 | **0.007** |
|  | b(x^3^) |  |  |  |  | 0.019 | **0.035** |  |  |  |  | 0.021 | **0.018** |
| HC (cm) | Constant | 99.700 | <0.001 | 97.465 | <0.001 | 95.279 | <0.001 | 102.221 | <0.001 | 99.074 | <0.001 | 98.000 | <0.001 |
|  | b(x) | -0.316 | **<0.001** | 0.420 | 0.189 | 1.719 | **0.022** | -0.136 | 0.088 | 0.860 | **0.030** | 1.455 | 0.134 |
|  | b(x^2^) |  |  | -0.057 | **0.018** | -0.280 | **0.019** |  |  | -0.074 | **0.010** | -0.172 | 0.247 |
|  | b(x^3^) |  |  |  |  | 0.012 | 0.056 |  |  |  |  | 0.005 | 0.502 |
| WrC (cm) | Constant | 17.639 | <0.001 | 17.574 | <0.001 | 17.300 | <0.001 | 16.320 | <0.001 | 15.722 | <0.001 | 15.596 | <0.001 |
|  | b(x) | -0.052 | **<0.001** | -0.031 | 0.552 | 0.132 | 0.275 | -0.007 | 0.545 | 0.183 | 0.001 | 0.252 | 0.054 |
|  | b(x^2^) |  |  | -0.002 | 0.667 | -0.030 | 0.124 |  |  | -0.014 | <0.001 | -0.026 | 0.203 |
|  | b(x^3^) |  |  |  |  | 0.001 | 0.138 |  |  |  |  | 0.001 | 0.561 |
| WHR | Constant | 0.923 | <0.001 | 0.917 | <0.001 | 0.901 | <0.001 | 0.965 | <0.001 | 0.965 | <0.001 | 0.929 | <0.001 |
|  | b(x) | -0.001 | 0.054 | 0.001 | 0.723 | 0.010 | 0.107 | -0.002 | **<0.001** | -0.002 | 0.364 | 0.018 | **0.006** |
|  | b(x^2^) |  |  | 0.001 | 0.431 | -0.002 | 0.082 |  |  | 0.001 | 0.992 | -0.003 | **0.001** |
|  | b(x^3^) |  |  |  |  | 0.001 | 0.107 |  |  |  |  | 0.001 | **0.001** |
| WHtR | Constant | 0.546 | <0.001 | 0.531 | <0.001 | 0.507 | <0.001 | 0.638 | <0.001 | 0.626 | <0.001 | 0.592 | <0.001 |
|  | b(x) | -0.002 | **<0.001** | 0.003 | 0.315 | 0.017 | **0.010** | -0.003 | <0.001 | 0.001 | 0.773 | 0.020 | **0.009** |
|  | b(x^2^) |  |  | 0.001 | 0.058 | -0.003 | **0.007** |  |  | 0.001 | 0.214 | -0.003 | **0.004** |
|  | b(x^3^) |  |  |  |  | 0.001 | **0.018** |  |  |  |  | 0.001 | **0.007** |
| Body fat (%) | Constant | 52.078 | <0.001 | 52.377 | <0.001 | 57.262 | <0.001 | 48.592 | <0.001 | 48.981 | <0.001 | 45.552 | <0.001 |
|  | b(x) | -0.287 | **0.016** | -0.386 | 0.513 | -3.332 | **0.036** | -0.017 | 0.876 | -0.141 | 0.787 | 1.730 | 0.158 |
|  | b(x^2^) |  |  | 0.008 | 0.863 | 0.527 | **0.046** |  |  | 0.009 | 0.807 | -0.295 | 0.110 |
|  | b(x^3^) |  |  |  |  | -0.028 | **0.046** |  |  |  |  | 0.015 | 0.092 |
| FMI (kg/m^2^) | Constant | 6.617 | <0.001 | 6.404 | <0.001 | 6.338 | <0.001 | 8.370 | <0.001 | 8.231 | <0.001 | 8.312 | <0.001 |
|  | b(x) | -0.017 | 0.692 | 0.054 | 0.799 | 0.094 | 0.869 | -0.042 | 0.369 | 0.002 | 0.991 | -0.042 | 0.938 |
|  | b(x^2^) |  |  | -0.006 | 0.733 | -0.013 | 0.895 |  |  | -0.003 | 0.841 | 0.004 | 0.962 |
|  | b(x^3^) |  |  |  |  | 0.001 | 0.940 |  |  |  |  | 0.001 | 0.927 |

b: regression coefficients. BMI= Body Mass Index, WC= Waist Circumferences, HC= Hip Circumferences, WrC= Wrist Circumferences, WHR= Waist to Hip Ratio, WHtR= Waist to Height Ratio, FMI=Fat Mass Index

Table S3. The binary logistic regression models of associations of anthropometric data, body fat percentage and fat mass index with sleep hours as a binary variable^+^ in both gender

| Model | Dependent variable | Independent variable | Male | | | | Female | | | |
| --- | --- | --- | --- | --- | --- | --- | --- | --- | --- | --- |
|  |  |  | OR | 95%CI | | P-value | OR | 95%CI | | P-value |
|  |  |  |  | L | U |  |  | L | U |  |
| Model 1 | BMI | sleep | **1.367** | 1.201 | 1.556 | **<0.001** | **1.249** | 1.114 | 1.400 | **<0.001** |
| Model 2 | (≥25) | sleep | **1.275** | 1.118 | 1.455 | **<0.001** | **1.219** | 1.084 | 1.370 | **0.001** |
| Model 3 |  | sleep | **1.299** | 1.136 | 1.485 | **<0.001** | **1.325** | 1.175 | 1.494 | **<0.001** |
| Model 1 | WC | sleep | **1.288** | 1.123 | 1.478 | **<0.001** | **1.237** | 1.013 | 1.511 | **0.037** |
| Model 2 | (Male>94 | sleep | **1.171** | 1.017 | 1.348 | **0.028** | 1.140 | 0.931 | 1.396 | 0.205 |
| Model 3 | Female>80) | sleep | **1.188** | 1.031 | 1.370 | **0.018** | **1.233** | 1.001 | 1.518 | **0.049** |
| Model 1 | HC | sleep | **1.311** | 1.145 | 1.500 | **<0.001** | **1.235** | 1.036 | 1.471 | **0.018** |
| Model 2 | (Male>100 | sleep | **1.224** | 1.067 | 1.404 | **0.004** | **1.251** | 1.045 | 1.499 | **0.015** |
| Model 3 | Female>90) | sleep | **1.243** | 1.082 | 1.429 | **0.002** | **1.357** | 1.128 | 1.633 | **0.001** |
| Model 1 | WrC | sleep | **1.411** | 1.234 | 1.612 | **<0.001** | **1.137** | 1.012 | 1.277 | **0.031** |
| Model 2 | (Male>17.5 | sleep | **1.314** | 1.147 | 1.504 | **<0.001** | 1.115 | 0.991 | 1.254 | 0.071 |
| Model 3 | Female>15.7) | sleep | **1.326** | 1.156 | 1.520 | **<0.001** | **1.191** | 1.055 | 1.344 | **0.005** |
| Model 1 | WHR | sleep | 1.076 | 0.946 | 1.225 | 0.264 | **1.384** | 1.101 | 1.738 | **0.005** |
| Model 2 | (Male≥0.90 | sleep | 0.971 | 0.849 | 1.111 | 0.668 | 1.245 | 0.987 | 1.570 | 0.064 |
| Model 3 | Female>0.85) | sleep | 0.985 | 0.860 | 1.128 | 0.828 | **1.307** | 1.031 | 1.656 | **0.027** |
| Model 1 | WHtR | sleep | **1.198** | 1.049 | 1.369 | **0.008** | **1.441** | 1.128 | 1.841 | **0.003** |
| Model 2 | (>0.5) | sleep | 1.075 | 0.937 | 1.233 | 0.305 | **1.310** | 1.023 | 1.679 | **0.033** |
| Model 3 |  | sleep | 1.093 | 0.951 | 1.255 | 0.211 | **1.421** | 1.101 | 1.835 | **0.007** |
| Model 1 | Body fat (%) | sleep | 1.179 | 0.897 | 1.549 | 0.239 | 1.210 | 0.985 | 1.485 | 0.069 |
| Model 2 | (Male ≥25 | sleep | 1.017 | 0.766 | 1.349 | 0.909 | 1.126 | 0.914 | 1.388 | 0.266 |
| Model 3 | Female ≥ 32) | sleep | 1.062 | 0.797 | 1.416 | 0.681 | **1.241** | 1.001 | 1.538 | **0.049** |
| Model 1 | FMI | sleep | 1.092 | 0.907 | 1.316 | 0.353 | 0.950 | 0.803 | 1.124 | 0.550 |
| Model 2 | (Male>7 | sleep | 1.084 | 0.897 | 1.308 | 0.404 | 0.887 | 0.748 | 1.053 | 0.171 |
| Model 3 | Female>7.9) | sleep | 1.085 | 0.898 | 1.311 | 0.400 | 0.924 | 0.777 | 1.100 | 0.374 |

Data presented as odds ratio (95%Confidence intervals). P-value reported as ^a^ <0.001, ^b^ <0.01, ^c^ <0.05 and significant results are bolded. BMI= Body Mass Index, WC= Waist Circumferences, HC= Hip Circumferences, WrC= Wrist Circumferences, WHR= Waist to Hip Ratio, WHtR= Waist to Height Ratio, FMI= Fat Mass Index. Model 1: unadjusted, Model 2: Adjusted with age, CVD history, socio-economic index, Model 3: adjusted with age, CVD history, socio-economic index and physical activity (MET score), dietary intake (energy intake Kcal/day). ^+^sleep considered as a binary variable in the model (Sleep hours<8 =1)

Table S4. The correlation between anthropometric and body fat composition indices. (N=4661)

|  | | BMI (kg/m^2^) | | WC (cm) | | HC (cm) | | WrC (cm) | | WHR | | WHtR | |
| --- | --- | --- | --- | --- | --- | --- | --- | --- | --- | --- | --- | --- | --- |
|  |  | r | P-value | r | P-value | r | P-value | r | P-value | r | P-value | r | P-value |
| Rt Leg | Fat mass | 0.115 | <0.001 | 0.137 | <0.001 | 0.111 | <0.001 | 0.034 | 0.020 | 0.107 | <0.001 | 0.137 | <0.001 |
|  | Fat-free mass | 0.030 | 0.039 | 0.010 | 0.504 | 0.048 | 0.001 | 0.112 | <0.001 | -0.043 | 0.003 | -0.034 | 0.019 |
| Lt Leg | Fat mass | 0.114 | <0.001 | 0.137 | <0.001 | 0.111 | <0.001 | 0.035 | 0.018 | 0.108 | <0.001 | 0.136 | <0.001 |
|  | Fat-free mass | 0.035 | 0.018 | 0.015 | 0.307 | 0.051 | <0.001 | 0.110 | <0.001 | -0.038 | 0.009 | -0.027 | 0.062 |
| Rt Arm | Fat mass | 0.140 | <0.001 | 0.155 | <0.001 | 0.145 | <0.001 | 0.074 | <0.001 | 0.097 | <0.001 | 0.142 | <0.001 |
|  | Fat-free mass | 0.019 | 0.187 | -0.001 | 0.951 | 0.034 | 0.021 | 0.104 | <0.001 | -0.045 | 0.002 | -0.042 | 0.004 |
| Lt Arm | Fat mass | 0.144 | <0.001 | 0.159 | <0.001 | 0.149 | <0.001 | 0.079 | <0.001 | 0.098 | <0.001 | 0.144 | <0.001 |
|  | Fat-free mass | 0.028 | 0.056 | 0.008 | 0.566 | 0.042 | 0.004 | 0.105 | <0.001 | -0.038 | 0.009 | -0.032 | 0.028 |
| Trunk | Fat mass | 0.130 | <0.001 | 0.153 | <0.001 | 0.142 | <0.001 | 0.087 | <0.001 | 0.098 | <0.001 | 0.130 | <0.001 |
|  | Fat-free mass | 0.027 | 0.061 | 0.008 | 0.567 | 0.041 | 0.006 | 0.106 | <0.001 | -0.037 | 0.012 | -0.034 | 0.022 |
| Total | Fat mass | 0.134 | <0.001 | 0.157 | <0.001 | 0.139 | <0.001 | 0.070 | <0.001 | 0.109 | <0.001 | 0.143 | <0.001 |
|  | Fat-free mass | 0.029 | 0.049 | 0.009 | 0.546 | 0.044 | 0.003 | 0.109 | <0.001 | -0.040 | 0.006 | -0.034 | 0.020 |
|  | Body fat (%) | 0.029 | 0.049 | 0.009 | 0.546 | 0.044 | 0.003 | 0.109 | <0.001 | -0.040 | 0.006 | -0.034 | 0.020 |
|  | FMI (kg/m^2^) | 0.185 | <0.001 | 0.187 | <0.001 | 0.141 | <0.001 | -0.028 | 0.057 | 0.162 | <0.001 | 0.255 | <0.001 |

Rt: right, Lt: Left, Fat mass and Fat-free mass unit: Kg, BMI= Body Mass Index, WC= Waist Circumferences, HC= Hip Circumferences, WrC= Wrist Circumferences, WHR= Waist to Hip Ratio, WHtR= Waist to Height Ratio, FMI=Fat Mass Index, r: Pearson correlation coefficient
